# Sorption of Fluoride and Bacterial Disinfection Property of Biosynthesized Nanofibrous Cellulose Decorated Ag–MgO–Nanohydroxyapatite Composite for Household Water Treatment

**DOI:** 10.3390/polym14050890

**Published:** 2022-02-23

**Authors:** Wasiu B. Ayinde, Mugera W. Gitari, James A. Smith, Amidou Samie

**Affiliations:** 1 Environmental Remediation and Nano Sciences (EnviReN), Department of Geography and Environmental Sciences, Faculty of Science, Engineering and Agriculture, University of Venda, Private Bag X5050, Thohoyandou 0950, South Africa; twasiu33@gmail.com; 2 School of Chemistry and Material Sciences, Technical University of Kenya, Haile Selassie Avenue, P.O. Box 52428, Nairobi 00200, Kenya; 3 Engineering Systems and Environmet, School of Engineering and Applied Sciences, University of Virginia, P.O. Box 400747, Charlottesville, VA 22904, USA; jas9e@virginia.edu; 4 Molecular Parasitology and Opportunistic Infections Program, Department of Biochemistry and Microbiology, Faculty of Science, Engineering, and Agriculture, University of Venda, Private Bag X5050, Thohoyandou 0950, South Africa; samie.amidou@univen.ac.za

**Keywords:** bioreduction, core–shell nanocomposite, cellulose nanocomposite, water purification

## Abstract

An innovative and sustainable approach to integrating modified Ag–MgO–nanohydroxyapatite on a nanofibrous cellulose template (CNF-AgMgOnHaP) as a multifunctional adsorbent via a hydrothermal bioreduction route using *Citrus paradisi* peel extract was developed and examined. The surface morphology and mineralogical properties of CNF-AgMgOnHaP by UV–vis spectroscopy, SEM-EDS, XRD, FTIR, TEM, and BET techniques are reported. Batch fluoride sorption studies and its disinfection potential against common bacteria in surface water were evaluated. The results showed the successful synthesis of a modified multistructural CNF-AgMgOnHaP composite with an improved BET surface area of 160.17 m^2^/g. The sorption of fluoride by the adsorbent was found to strongly depend on the different sorption conditions with a maximum F^−^ sorption capacity of 8.71 mg/g at 303 K, and pH of 5 with 0.25 g dosage at 10 min contact time (25 ± 3 °C). Equilibrium fluoride sorption onto the CNF-AgMgOnHaP was best described by the Freundlich isotherm model across all the operating temperatures. The overall kinetic results showed that the adsorption mechanisms not only depend on using the pseudo-second-order process but are also governed by the mass transfer of the adsorbate molecules from the external surface onto the pores of the adsorbent. The thermodynamic parameters revealed that the adsorption process of F^−^ onto CNF-AgMgOnHaP was endothermic and spontaneous at the sorbent/solution interface. The synthesized composite also provides some antibacterial activity against common infectious microbes from contaminated drinking water. The overall results suggested that the CNF-AgMgOnHaP nanocomposite possesses the potential for the simultaneous decontamination of pollutants and microbes in drinking water.

## 1. Introduction

Water, according to the United Nations World Water Development Reports [1], is at the core of sustainable development. It is widely acknowledged that providing safe and clean water to communities is the most crucial part of enhancing the wellbeing of people. The lack of centralized treated water supply systems means the rural communities of most developing and sub-Saharan African countries rely on unsafe surface and groundwater sources for their domestic water supply [2,3]. The majority of untreated waters in these rural communities are prone to toxic inorganic metal ions and infectious microbes introduced by natural and anthropogenic activities, resulting in death from water related diseases [4].

Fluoride concentration is an important toxic inorganic contaminant in groundwater resources from the standpoint of public health around the world. Excess fluoride in drinking water is estimated to be a problem in many countries around the world, with significant health effects and morbidity in many regions depending on geographical and economic status [5,6,7]. Fluoride, below the recommended limit of 1.5 mg/L, is an important component in preventing tooth cavities, and facilitating the mineralization of bone, dental enamel, and arduous tissues in humans [8,9,10]. However, in excess, it can be detrimental to human health, leading to dental or crippling skeletal fluorosis [8,11,12]. Besides fluoride epidemics, most acute water related diseases are often associated with the consumption of infectious microbes from contaminated water, which are responsible for about 2.2 million deaths yearly in developing countries [8,13,14]. The disinfection of microbial contamination in these water sources has proven effective against the adverse effects on human health. The disinfection of these microbiological hazards from contaminated water using the conventional chlorination method is rapidly becoming a major challenge, because of microbial resistance and the formation of detrimental reproductive and carcinogenic disinfection byproducts (DBPs) [15,16]. The importance of removing these toxic substances and water disinfection through innovative and sustainable technology cannot be overstated, for improved water quality and ecology. The remediation techniques used are based on the sorption principle as well as chemical cell death by disrupting the cell walls of microbes, particularly at the point of use [17,18]. Many of the materials developed, to date, based on such technologies, as well as their modes of operation and mechanisms, have been reviewed and reported [7,17,18,19,20]. However, challenges, such as high operating costs, high maintenance, low adsorption capacity, incomplete pollutant removal, and toxic sludge release [10,21,22], posed by these technologies have limited their potential field applications.

To overcome these drawbacks, intense efforts are now being directed toward the development of low cost, high selectivity and capacity, readily available, ecofriendly, and sustainable multifunctional biopolymeric reinforced composites, to address these recurring waterborne epidemics. In accordance with the World Health Organization’s (W.H.O.) technological sustainability, biopolymeric reinforced composites provide a solution for economically and ecologically viable multifunctional water purification techniques, to adequately remediate these pollutants in contaminated water resources for use in household water treatment devices.

Nanocellulose has been receiving considerable interest in preparing new composite materials that selectively target toxic metal ions in water because of its surface functionalizable hydroxyl, inherent hydrogen bonds, and van der Waal forces, which, in synergy, may enhance the adsorption capacity of the targeted chemical species [23,24]. Its many remarkable properties, such as mechanical strength, biodegradability, nontoxicity, and renewability, serve as an edge for its application in water treatment, compared to other equivalent sorbent materials [25]. We have recently reported the systemic development and effectiveness of antimicrobial based biogenic nanoparticles and multifunctional metal/metal oxides composites for fluoride and pathogens decontamination from water [26,27,28].

This research is a continuation of the integration, demonstrating the multifunctional properties of biopolymers as a metal based composite reinforcement; due to their exceptional characteristics that offer vast potential in remediating hazardous contaminants from aqueous solution. Therefore, the focus of this study was to isolate cellulose nanofiber from sawdust; biosynthesize a modified Ag-MgO nanoparticle using *Citrus paradisi* (Grapefruit red) peel extracts as a bioreducing, stabilization, and capping agent on nanohydroxyapatite nanocomposite. To create an improved multifunctional adsorbent by impregnating the Ag-MgOnHaP onto a cellulose nanofiber network, which is intended to simultaneously remediate toxic inorganic and microbial contaminants in drinking water. It is also envisaged that the fabricated adsorbent can be applied in household water treatment devices.

## 2. Materials and Methods

### 2.1. Chemicals and Reagents

All chemicals were of analytical reagent grade. Ca(NO_3_)_2_·4H_2_O, KH_2_PO_4_, Mg(NO_3_)_2_·6H_2_O, AgNO_3_, NaClO_2_, H_2_SO_4_, NaF, NaOH, and other chemicals used were obtained from Sigma-Aldrich (St. Louis, MO, USA) and supplied by Rochelle Chemicals, Johannesburg, South Africa. The raw material used in the preparation of cellulose nanofiber in this work is sawdust/wood chips (waste product of woodworking operations) and the peel of *Citrus paradisi* (Grapefruit red). Raw sawdust was collected from the School of Agriculture, University of Venda, South Africa. Ultrapure water from Ultrapure Milli-Q water S.A.S (Molsheim, France) (18.2 MΩ/cm) was used in the preparation and dilution of standards throughout the experiment.

### 2.2. Preparation of Adsorbent

#### 2.2.1. Preparation of *Citrus Paradisi* Peel Extract

The fruit peels of *Citrus paradisi* (Grapefruit red) were removed, cleaned thoroughly using Ultrapure Milli-Q water S.A.S (Molsheim, France) (18.2 MΩ/cm) to remove any dust particles adhering to the surface and cut to small pieces. A total of 30 g of the peel was added to 100 mL of Ultrapure water and boiled for 20 min at 70 °C. The extract was cooled and filtered through Whatman No.1 filter paper (Cytiva, Shrewsbury, MA, USA) and stored at 4 °C for further use.

#### 2.2.2. Preparation of Cellulose Nanofibers from Sawdust Biomass (CNF)

Extraction of cellulose nanofiber was carried out by a modified method for purification of sawdust through chemical (alkaline-acid) and mechanical (ultrasound) modes of treatment, as described by [29,30]. The sawdust was first dried in sunlight and then cut to small sizes using a domestic blending machine (PHILIP, 400W, HR 2103) and passed through a 100-mesh sieve. The ground pieces were further dried in a hot air oven overnight at 65 °C before the alkaline pretreatment. The pretreatment soaking was carried out in an alkali solution (4 wt.% NaOH solution) at room temperature for 3 h, followed by continuous washing with Ultrapure water (18.2 MΩ/cm). The obtained pretreated fibers were delignified by bleaching with a buffer solution (pH 4.5) of acetic acid 5% (*w*/*v*), sodium chlorite (NaClO_2_), and Ultrapure water boiled for 3 h at 70 °C with a pulp to liquor ratio of 1:1. The mixture was allowed to cool and filtered and washed using excess Ultra-pure water (18.2 MΩ/cm). The bleaching process was repeated three times. The obtained delignified and hemicellulose free cellulose fibers were further subjected to acid hydrolysis using 8.5 M of sulphuric acid (fiber to liquor ratio of 1:10) for 1 h at 50 °C. The reaction mixture was stopped by washing several times (in 10-fold excess Ultrapure water), followed by centrifugation at 4500 rpm at room temperature for 30 min until the resultant supernatant liquor became turbid (pH between 5–6). The obtained suspension was stored in the refrigerator at 4 °C for further use. The resulting suspension (colloidal cellulose particles) was thereafter subjected to sonication for 45 min in an ice bath to obtain the nanofibers.

#### 2.2.3. Preparation of Nanohydroxyapatite (nHaP)

Synthetic nanohydroxyapatite (nHaP) was prepared as described by Poinern et al. [31], by reacting 40 mL of 0.32 M Ca(NO_3_)_2_·4H_2_O with 60 mL of 0.19 M KH_2_PO_4_ solution under continuous stirring with the Ca/P ratio maintained at 1.67. Subsequently, aqueous NH_4_OH (25%) was added dropwise to adjust the pH value to 9, and the solution was stirred for 6 h and thereafter left to age for 24 h at room temperature. During the mixing process, the pH value was continually checked and maintained at 9 (using NH_4_OH). The composite solution was thereafter subjected to ultrasound agitation at 100% amplitude (0.5 cycles) for 1 h. The product obtained after the sonication was filtered and dried at 40 °C in an oven for 24 h before being ground into a fine powder.

#### 2.2.4. Synthesis Conditions of Cellulose–Nanofiber–AgMgOnanohydoxyapatite

The cellulose–nanofiber–AgMgOnanohydoxyapatite composite was synthesized by incorporating nanohydroxyapatite bound to Ag-MgO nanoparticle into a cellulose nanofiber matrix via bioreduction and in situ precipitation method at room temperature. For this, 10 g (*w/w* %) of the obtained colloidal cellulose suspension from sawdust (CNF) was added to a mixture of solutions containing 40 mL of aqueous Citrus paradisi peel extracts, 60 mL 1 mM AgNO_3_, and 20 mL 0.1 M Mg(NO_3_)_2_·6H_2_O. The solution was mixed under continuous stirring for 12 h at 40 °C. The bioprocess seed growth kinetics of the nanocomposite through color variation was monitored using UV–Vis spectroscopy. To this continuously stirred bioreduced reaction mixture (CNF-AgMgO), 5 g of nHaP was dispersed into the mixture and was agitated on a magnetic stirrer (Heidolph (Schwabach, Germany)) for 8 h to achieve homogenous mixing. The mixture was filtered and oven dried at 60 °C for 24 h, and then ground to obtain the cellulose–nanofiber–AgMgOnHaP (CNF-AgMgOnHaP). The synthesis routes were repeated by varying the colloidal cellulose suspension loading within the CNF–AgMgOnHaP composite from 10 to 100 (*w/w* %) to evaluate the optimum loading weight percentage required for optimum defluoridation efficiency.

### 2.3. Instruments for Material Characterization

The surface morphological, elemental analysis, and physicochemical compositions of the synthesized sorbent were assessed using a scanning electron microscope (SEM) (FEI Nova, Brno, Czechoslovakia Republic) with an FEI Nova NanoSEM 230 with a field emission gun equipped with an Oxford Xmax SDD detector operating at an accelerating voltage of 20 kV for the EDS detector (Oxford X-Max with INCA software). The ALPHA Fourier Transform Infrared spectrum (4000–400 cm^−1^) equipped with ATR-Diamond (Bruker, Karlsruhe, Germany) was used to obtain the infrared spectrum of the sorbent. Bruker-D8 Powder Diffractometer (Bruker D8 Advance, Cu-K radiation, wavelength 1.54443Å Lynx-eye XE detector) with a theta-theta goniometer X-ray diffraction (XRD) technique was employed to examine the sorbent structural phase modification. The CNF-AgMgOnHaP composite kinetic growth rate was characterized using UV-Visible Spectrophotometers (220–600 nm), (SPECTROstar Nano/BMG LABTECH). Transmission electron microscopy (TEM) images were taken using an FEI Tecnai20 (Hillsboro, OR, USA) equipped with a LaB6 emitter, operating at 200 kV and fitted with a Gatan Tridiem GIF with a 2k × 2k CCD camera. Images were collected using the Digital Micrograph suite of programs for size and shape. The surface area, pore area, and pore volume of the synthesized nanocomposite were measured using nitrogen adsorption Brunauer–Emmett–Teller (BET) surface area and porosity analyzer (Micromeritics ASAP2020). The adsorption–desorption plots were used to calculate the specific surface area. UP400S (400 W at 24 kHz power control amplitude 20–100% ultrasonic device with ultrasonic horn H14) from Hielscher Ultrasonic was used for sonicating. The surface interaction and composition of the fluoride loaded sorbent were studied by the X-ray Photoelectron Spectrometer Microprobe (XPS) (Thermo Scientific ESCAlab 250Xi) (Waltham, MA, USA), with a monochromatic Al Kα X-ray source (1486.7 eV). The high resolution scans were conducted according to the peak being examined with a pass energy of 20 eV (Pressure < 8–10 mBar) and spot size of 900 μm with a wide survey scan between 0–1300 eV.

### 2.4. Batch Fluoride Adsorption Procedure

Batch fluoride sorption studies were performed and evaluated with the synthesized CNF-AgMgOnHaP for the removal of fluoride in simulated and field groundwater. The effects of contact time, pH, adsorbent dose, initial adsorbate concentration, and coexisting ions on the equilibrium adsorption capacity were optimized. Standard stock fluoride ion solution (1000 mg/L) was prepared by dissolving 2.210 g NaF into 1000 mL of Ultrapure water under ambient conditions. The desired fluoride solution was prepared by the dilution of the standard stock solution. Batch adsorption experiments were carried out by mixing 0.225 g of CNF-AgMgOnHaP with 50 mL of 10 mg/L F^−^ solution. The mixture was shaken thoroughly using a reciprocating shaker (STUART SSL2) (Staffordshire, UK) at 250 rpm. The solution was then filtered, and the residual fluoride ion concentration was determined. F^−^ and pH were determined using a fluoride ion selective electrode (9609 BNWP) (Orion) (Waltham, MA, USA) coupled to an ISE/pH/EC electrode (Thermo SCIENTIFIC-ORION VERSA STAR Advanced Electrochemistry meter fluoride ion selective electrode) calibrated with four fluoride standards containing TISAB III at the volume ratio of 1:10, as with the samples. The pH values of the solution were adjusted by 0.1 mol/L HCl or NaOH. The effect of coexisting anions on the defluoridation efficiency of the adsorbent was evaluated at an anion concentration of 10–30 mg/L in a 10 mg/L fluoride solution. The adsorption isotherm experiments were conducted by varying fluoride concentrations within the range of 5–100 mg/L in a constant temperature water bath shaker (EcoBath) (Kyonggi, Korea) in the temperature range of 303, 313 and 323 K. Adsorption isotherms and kinetic models were adopted and used to model adsorption data. All the experiments were conducted in triplicate, and the mean of the results was computed. Equations (1) and (2) were used to determine the percentage fluoride removal and adsorption capacity, *q_e_* (mg/g) of the adsorbent.

(1) $$\%\;\mathrm{Fluoride}\;\mathrm{Adsorption}\;=\;\frac{\left(C_{o}\;-\;C_{e}\right)}{C_{o}}\;\times \;100$$

(2) $$q_{e}\;=\;(C_{o}\;-\;C_{e})\;\times \;\frac{V}{m}$$

where *C_o_* is the initial F^−^ concentration (mg/L); *C_e_* is the F^−^ concentration at equilibrium (mg/L); *V* is the volume of solution (L), and *m* is the dried mass of the adsorbent (g).

### 2.5. Point of Zero Charge (pHpzc)

The pH at the point of zero charge of CNF-AgMgOnHaP was determined in 1 M, 0.1 M, and 0.01 M KCl solutions for consistency of results. The pH of solutions was adjusted to desired values by adding 0.1 M HCl or 0.1 M NaOH. The new pH, therefore, constituted the initial pH (pHi) of solutions. Aliquots of 25 mL of solutions were pipetted into 50 mL plastic bottles. A mass of 0.25 g of adsorbent was then weighed into each of the bottles. The bottles were corked and shaken inside a constant temperature water bath shaker at 150 rpm for 24 h. After equilibration, the equilibrium pH (pHe) of each mixture was quickly measured and recorded.

### 2.6. Regeneration of CNF-AgMgOnHaP Composite

The regeneration experiments were conducted to evaluate the reusability of the spent sorbent using 0.01 M NaOH and 0.1 M Na_2_CO_3_ solutions. In each case of the desorption process, 0.25 g of CNF-AgMgOnHaP composite was added to 50 mL of 10 mg/L fluoride solution. The fluoride loaded adsorbent was agitated separately with 50 mL of 0.01 M NaOH and 0.1 M Na_2_CO_3_ for 30 min, thereafter the adsorbent was filtered, and the filtrate was subsequently analyzed for residual fluoride. The collected adsorbent on the filter membrane was washed with Milli-Q water and then dried at 80 °C for 3 h. The regenerated adsorbent was then reused for further fluoride removal for up to four regeneration cycles.

### 2.7. Antibacterial Evaluation of the Composite

The antibacterial efficacy of the biosynthesized CNF-AgMgOnHaP nanocomposite was determined qualitatively from the observed zone of inhibition (mm) using the standard agar well disc diffusion methods (Kirby Bauer disk diffusion test). The indicator strains used were *Escherichia coli* (ATCC 35218), *Staphylococcus aureus* (ATCC 33591), and *Klebsiella pneumonia* (ATCC 700603). Bacterial suspensions were prepared with a turbidity of 0.5 McFarland. A total of 50 μL of each strain was spread evenly on 20 mL of solidified and dried freshly prepared MHA agar plates. Wells were punctured at equidistance using sterile pipette tips. A total of 50 μL each of CNF-AgMgOnHaP was dispensed into the wells already inoculated with the bacterial cell suspension. The plates were incubated at 37 °C for 24 h and the diameter of the zone of growth inhibition around the different sample concentrations (1–10 mg/mL) was measured in millimeters (mm). The measured zones of inhibition were used to determine the antibacterial activities of the nanocomposite.

### 2.8. Statistical Tools

The computations were carried out using OriginPro 8.SR0 and Excel software. The best fit sorption models were analyzed using the linear and nonlinear analysis, adjusted correlation coefficient (*Adj. R*^2^), and chi-square analysis (*χ*2) were computed.

## 3. Results and Discussions

### 3.1. Effects of Cellulose Nanofiber Loading (wt.%) in CNF-AgMgOnHaP Adsorbent on Adsorption

The percentage weight effects of cellulose nanofibers in the CNF-AgMgOnHaP composite were evaluated for fluoride sorption capacity. This was carried out by contacting 0.225 g of the composite with 50 mL of 10 mg/L initial fluoride solution at 250 rpm for 30 min. Table 1 shows the results of percentage fluoride removal. The results suggests that the higher the CNF weight content, the more the hydroxyl groups present in the composite, resulting in more binding sites for the fluoride sorption capacity throughout the composite matrices.

### 3.2. Structural Morphological Analysis

#### 3.2.1. UV-Visible Study

**Figure 1:**
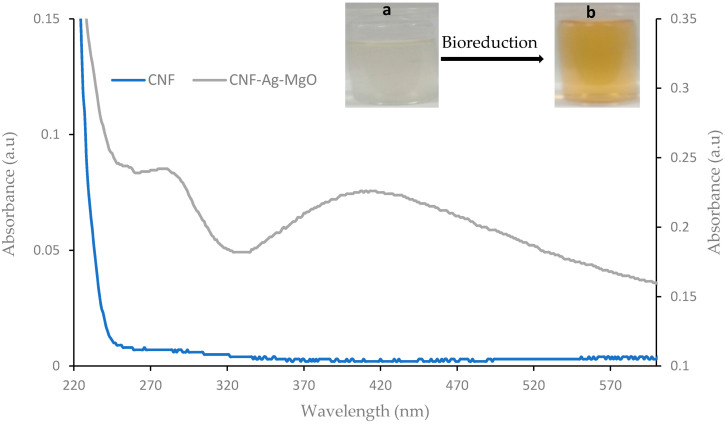
UV–Vis spectrum of CNF-AgMgO synthesized using *C. paradisi* peel extracts as a reducing agent at 40 ± 2 °C.

The successful impregnation of the Ag-MgO nanoparticles on the cellulose nanofiber matrix is shown in the UV–visible absorbance spectra (Figure 1). The bioreduction formation of the nanoparticles by aqueous peel extracts of *C. paradisi* is shown by a change in the color of the reaction solution from colorless to light yellow (inserted image in Figure 1). As shown in the spectra, no visible absorption peak was observed in the bare cellulose nanofiber (CNF); however, two different distinct peaks were observed in the CNF-AgMgO composite. An absorption band is observed in the low UV region range from 270 to 290 nm, typical of MgO, together with a broad band around 420 nm associated with the characteristic absorption of silver nanoparticles [32,33] The change in the reaction mixture color variation is attributed to the surface plasmon resonance of excited electrons and O^2−^ surface anions at the surface of Ag and MgO nanoparticles, respectively, in resonance with a light wave [34,35,36]. The availability and interaction of the bioactive functional species in the *C. paradisi* peel extracts and that of the cellulose hydroxyl groups with the silver ions may be responsible for the rate of bioreduction, as shown in the optical property of biomolecular capped Ag-MgO nanocomposite into the nanofiber [37,38]. Furthermore, the cellulose fiber matrix used during the synthesis, in addition to contributing as reducing agents, also provided good stability to the CNF-AgMgO composite, thereby preventing agglomeration resulting in uniform nucleation and growth conditions for impregnated nanoparticles [39,40].

#### 3.2.2. Electron Microscopic Analysis

**Figure 2:**
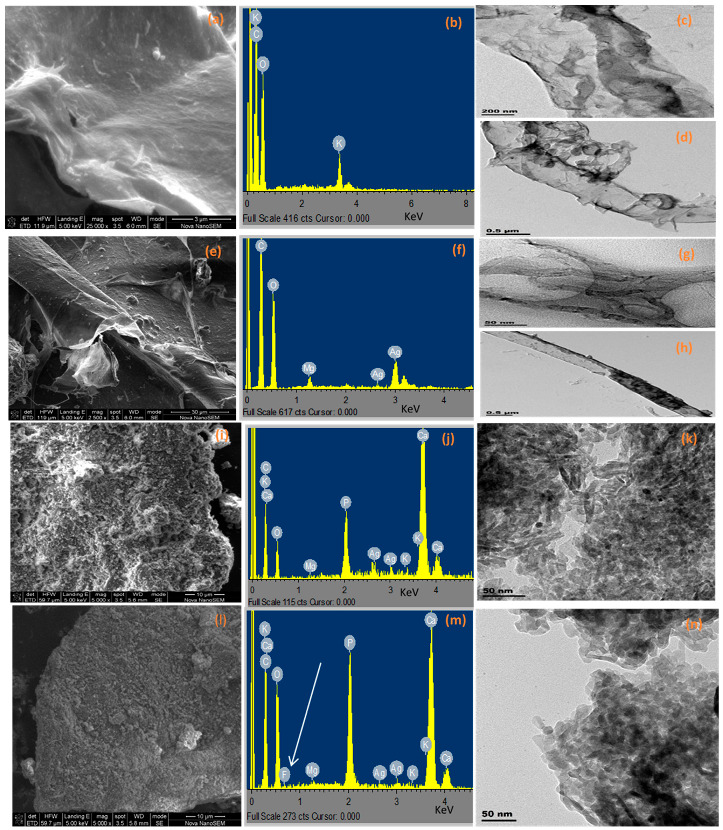
SEM, EDS and TEM of (**a**–**d**) CNF derived from sawdust waste; (**e**–**h**) CNF-AgMgO composite, (**i**–**k**) CNF-AgMgOnHaP adsorbent before fluoride sorption; (**l**–**n**) CNF-AgMgOnHaP adsorbent after fluoride sorption.

Figure 2a–n show the SEM images, different size distributions with associated EDS patterns, and the TEM micrographs of the systemically developed composite materials before and after fluoride sorption, respectively. The images of successfully extracted fine and uniform cellulose fibril structure from sawdust and its corresponding compositions are shown in Figure 2a–d. The typical elemental composition of the CNF derived from a sawdust waste constituent is shown on the EDS spectrum (Figure 2b). The obtained fiber is elongated (Figure 2d) with a wide width diameter of about 0.5 μm. Figure 2e showed the SEM image of the synthesized bioreduced CNF-AgMgO nanocomposite from the *C. paradisi* peel extracts with its corresponding EDS (Figure 2f), revealing the Ag-MgO nanoparticles embedded in the cellulose fiber network. The TEM micrograph (Figure 2g,h) displayed a weblike layout surface structure with a thin, long threadlike individual size of about 30–50 nm in width. It is important to note that the morphologies of CNF and the bioreduced CNF-AgMgO nanocomposite maintain the same elongated, rod like structure, confirming the nanofibrous morphology and the impregnation of the nanoparticles on the surface of the CNF. Figure 2i–n show the different structural modifications of the nHaP impregnated CNF-AgMgO (CNF-AgMgOnHaP) and its corresponding defluoridated CNF-AgMgOnHaP composites. Figure 2i,l represent the SEM images of CNF-AgMgOnHaP and fluoride sorbed CNF-AgMgOnHaP, respectively, and the transformed granulated aggregation of nanoparticles on the cellulose fiber of both composites can be observed. This could be attributed to the very irregular surface of the incorporated nanohydroxyapatite powder after drying the composite [41]. The presence of the inherent elemental distribution around the cellulose matrix of the synthesized CNF-AgMgOnHaP adsorbent is shown in Figure 2j, and the aggregation of the adsorbent at a size distribution range of 50 nm is presented in Figure 2k. The corresponding morphological structure of the sorbed fluoride-CNF-AgMgOnHaP composites is presented in Figure 2l–n. The presence of elemental fluorine in the EDX spectrum (Figure 2m) suggested the potential of the material towards deflouridation.

#### 3.2.3. FTIR Spectroscopy

**Figure 3:**
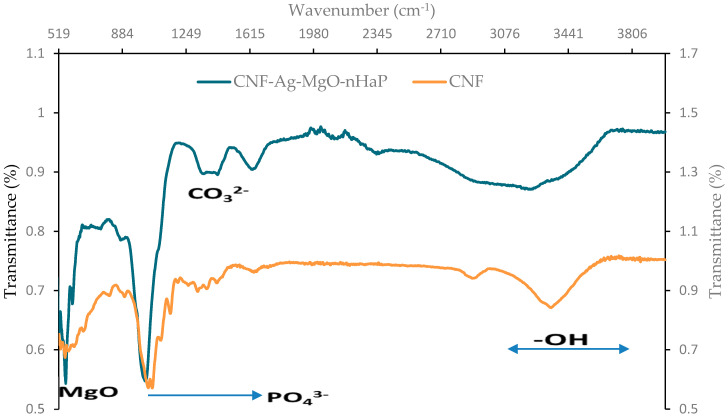
The FTIR spectra of CNF and CNF-AgMgOnHaP adsorbent.

Figure 3 shows the transition and bond formation between the cellulose nanofiber from the source (sawdust) and the CNF-AgMgOnHaP matrices. The major characteristic functional group peaks observed in the CNF are found at 3343, 2904, 1640, 1425–1433, 1320–1335, 1162, 1054–1052, and 895–585 cm^−1^, while the spectrum of the CNF-AgMgOnHaP composite showed adsorption bands at 3229, 1634, 1432–1353, 1017, 893, 595, and 558 cm^−1^. In both spectrums, the presence of strong stretching vibration derived from the -OH group is seen at 3343 cm^−1^ at the CNF, which became broader and shifted to the lower range of 3230–3229 cm^−1^ in the CNF-AgMgOnHaP composite. The broadness of the band in the composite may be attributed to the contribution of OH^−^ groups in the matrix through the oxidation of the cellulose in the bioreduction of the impregnated nanoparticles across the composite [42]. The bands at 2904, 1320–1335, and 1140–1117 cm^−1^ represent C-H symmetrical stretching, CH_2_ wagging vibration, an in the plane -CH bending mode, and stretching vibration from C-O and C-O-C groups, respectively, characteristics of natural fiber [43]. Characteristic peaks at around 1436–1450, 1032–1020, 912–656, 598–474 cm^−1^ assigned to a vibrational mode of CO_3_^2−^, the asymmetric phosphate group, PO_4_^3−^ stretching and bending modes, and Mg-O-Mg deformation of the Mg-O absorption, respectively, featured in the CNF-AgMgOnHaP composite spectra [44,45,46]. Therefore, the result obtained from the FTIR spectrometry suggests the presence of nanohydroxyapatite (nHaP) and MgO species impregnation within the CNF matrix, with a considerable amplification in intensities.

#### 3.2.4. Specific Surface Area by Brunauer–Emmett–Teller (BET)

**Figure 4:**
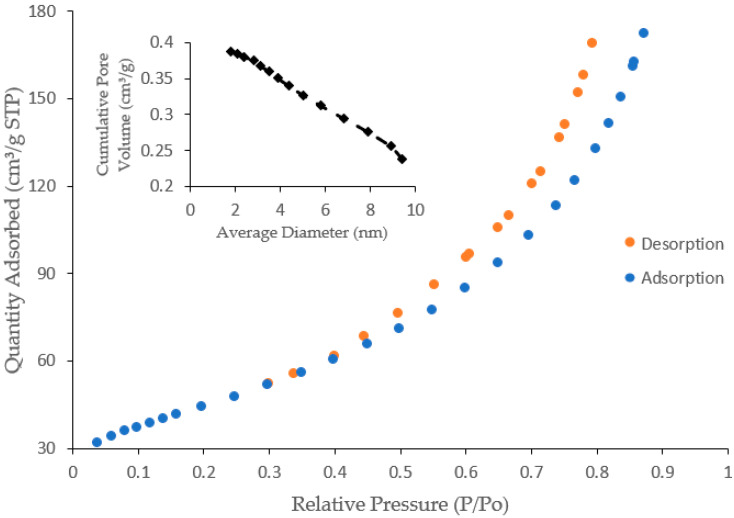
BET Nitrogen adsorption–desorption isotherms and pore size distribution curve.

The specific surface area of the CNF-AgMgOnHaP adsorbent was examined through the BET nitrogen adsorption–desorption measurements at an ambient temperature of 22 °C and the results tabulated in Figure 4 and Table 2, respectively. As shown in Figure 4, the composite isotherms followed Type I classification. However, the composite average pore width was found to be 9.5541 nm, demonstrating the characteristics of micro-mesoporous materials [47]. The CNF-AgMgOnHaP is characterized by a BET surface area of 160.17 m^2^/g with a micropore and mesopore area of 5.46 m^2^/g and 196.12 m^2^/g, respectively. The increase in the surface area of CNF-AgMgOnHaP composite is attributed to an increase in the availability of many binding functional groups within the matrix, as compared to the specific surface area of soft cellulose pulp (1 and 4 m^2^/g) [48,49]; apatite materials (36.00 m^2^/g; 69.68 m^2^/g) [50,51]; as well as silver and MgO nanoparticles, respectively (56.9 m^2^/g and 114.03 m^2^/g) [52,53].

#### 3.2.5. XRD Analysis

**Figure 5:**
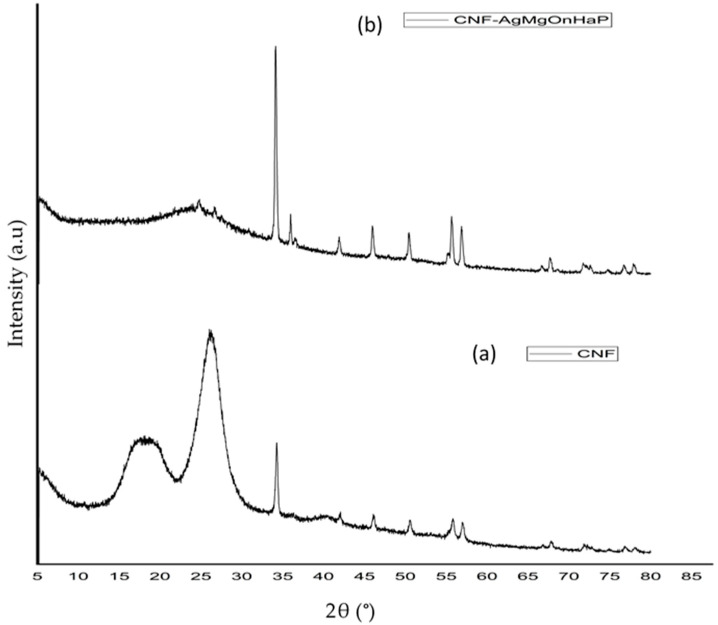
XRD patterns of (**a**) CNF from sawdust (**b**) CNF-AgMgOnHaP composite.

Figure 5 displays the XRD patterns between the extracted CNF from sawdust and the CNF-AgMgOnHaP composite. The XRD of the extracted CNF (Figure 5a) depicts intensive XRD diffractograms 2θ peaks at ∼16, ~22.9 (200 planes), and ~34°, which are related to a typical peak pattern structure of the native cellulose I structure (ICDD: PDF database 1999 no: 00-003-0289) [54,55] However, a change in the structural orientation was observed in the CNF-AgMgOnHaP composite diffractogram (Figure 5b). The crystalline phases and intensities observed in the adsorbent XRD spectrum (b) illustrate the presence of different components in the composite compared to that of the CNF (spectrum a). The diffraction peaks at 16.89 (010), 26.33 (210), 32.15 (211), 39.7 (111), 44.4° (200), 46.65, and 49.89° (213) in 2θ revealed the presence of hexagonal carbonate-hydroxyapatite (entry no: 96-9003-551) [56]; triclinic crystal system of silver attached to the phosphate groups (entry no: 96-100-8002) [57]. Characteristic crystal structure peaks for monoclinic Mg within the apatite phase at the 2θ value of 19.36 (100), 31.52 (020), and 53.49° (031) (entry no: 96-231-0425) [58] were also identified (Figure 5b). The typically defined cellulose peaks were also visible in the patterns of the composites. These results suggest that the difference observed in the surface crystallinity between these materials arises because of the impregnated nanoparticles on the CNF polymer matrix morphology.

## 4. Batch Fluoride Adsorption Experimental Results

### 4.1. Effect of Contact Time

**Figure 6:**
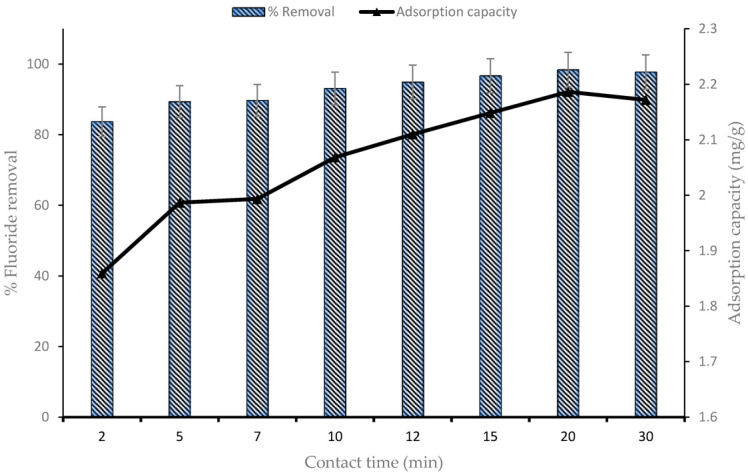
Effect of contact time on CNF-AgMgOnHaP adsorbent. (Experimental conditions: dosage, 0.225 g; volume, 50 mL; initial concentration, 10 mg/L; pH, neutral; temperature, 298 K and shaking speed, 250 rpm).

The F^−^ uptake using the CNF-AgMgOnHaP adsorbent was investigated as a function of contact time, between 0–30 min, using a 0.225 g adsorbent dose per 50 mL of 10 mg/L initial fluoride concentration (Figure 6). The fluoride sorption process increased sharply as the time increased, within the first 10 min of contact time with about 93% of F^−^ removal. Subsequently, the adsorption rate became slower with increasing contact time (10–20 min), with no appreciable difference in the percent fluoride removal until complete saturation was achieved. Thus, a contact time of 10 min was chosen as the optimum time for fluoride sorption by the CNF-AgMgOnHaP. The rate of fluoride removal within the rapid equilibration time was due to the presence of free and abundant binding sites across the adsorbent surface. This rapid interaction at the earlier stage suggests the presence and availability of a high amount of hydroxyl groups (supported by FTIR (Figure 3)) within the composite, which leads to high F^−^ sorption because of the easy exchange of the fluoride ions with the CNF-AgMgOnHaP surface hydroxyl groups. Furthermore, the improved surface area and wider pore dimensions also accelerated the high fluoride sorption accessibility to the binding sites on the adsorbent surface.

### 4.2. Effect of Sorbent Dosage

**Figure 7:**
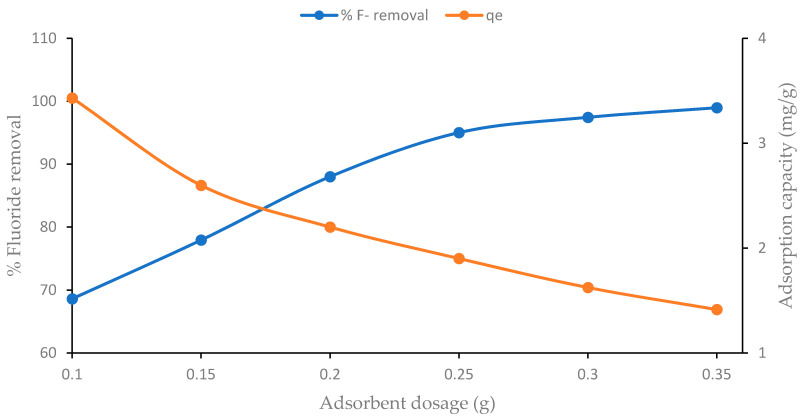
Effect of sorbent dose on the removal of F^−^ ions by CNF-AgMgOnHap composite (contact time of 10 min, initial F^−^ concentration of 10 mg/L at 50 mL solution volume, pH of 5, shaking speed of 250 rpm).

The adsorption efficiency of the F^−^ ion by the CNF-AgMgOnHaP dosage was investigated and the result is shown in Figure 7. The F^−^ removal efficiency increases with an increase in adsorbent dosage (0.1 to 0.35 g) attaining a constant optimum removal at 0.35 g. This may be attributed to the fact that increasing the dose of CNF-AgMgOnHaP creates more vacant available surface binding sites favoring the fluoride sorption. In addition, the increased structural surface area and porosity of the synthesized adsorbent, as confirmed by the BET analysis (Table 2), may have contributed to the high fluoride sorption capacity.

### 4.3. Effect of pH and Surface Charges on Fluoride Sorption

**Figure 8:**
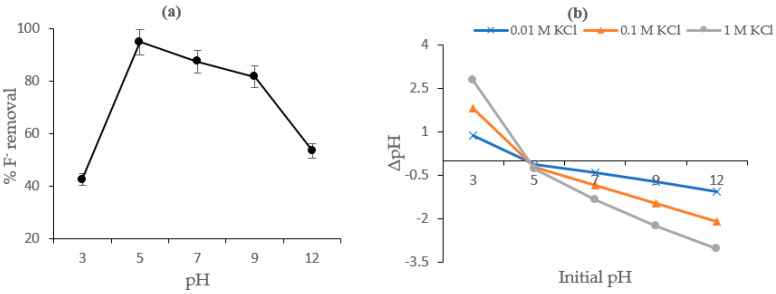
(**a**) Effect of initial solution pH; (**b**) pH_pzc_ of CNF-AgMgOnHaP (adsorbent dose: 0.25 g volume of solution: 25 mL, contact time: 24 h at 150 rpm).

The effect of initial solution pH (3–12) as it affects the surface charges of the CNF-AgMgOnHaP adsorbent in the removal of fluoride is shown in Figure 8a,b. It is clear from the plot (Figure 8a) that fluoride sorption capacity by the adsorbent was pH dependent and the maximum sorption percentage was observed at pH 5. Thereafter, the rate of removal dropped from 87% at pH 7 to 82% at pH 9, with significant adsorption reduction after pH 9, to pH 12 at 54%. This variation might be attributed to the changes within the solution pH environment which ultimately changes the surface charge and the protonation/deprotonation of the binding functional groups across the CNF-AgMgOnHaP adsorbent, as well as the solubility of fluoride ion species during the process [58,59]. The surface charges distributions of the CNF-AgMgOnHaP adsorbent in fluoride sorption at different initial solution pH values (3–12) were also evaluated by its point of zero charge (pHpzc). The pHpzc is defined as that pH value at which the net surface charge density of an adsorbent is zero [60]. As shown in Figure 8b, the pHpzc value of the CNF-AgMgOnHaP was at ~4.7, which is lower than the optimum pH value of 5. Consequently, the surface of the sorbent becomes negatively charged in the solutions at which the equilibrium pH was greater than pHpzc.

This means that there are abundant negative charges on the surface of CNF-AgMgOnHaP at pH > 4.7 which interact and replace the fluoride ions in solution. One of the main contributing surface groups for a high adsorption of F^−^ by the adsorbent is the availability of -OH groups in the cellulose and hydroxyapatite (recognized by FTIR in Section 3.2.3). It is known that both F and -OH are structurally isoelectronic, having a comparable ionic radius, and can easily replace one another through ligand exchange, which ultimately favors F^−^ anions’ removal with consequent high sorption capacity and selectivity [3,61]. Besides, the hydrolyzing agent in the isolation of the stabilized cellulose nanofiber, sulfuric acid, reacts with the cellulose surface hydroxyl groups to form sulfate half-esters, which also contributed to the negatively charged surface of the adsorbent, thus, the high adsorption of fluoride by CNF-AgMgOnHaP [62]. Therefore, the interaction between these surface groups and fluoride ions easily facilitates the high fluoride sorption capacity through an ion exchange mechanism, the formation of an ion pair, or through H bonding with the negatively charged surface. A similar pattern of surface exchange mechanisms of fluoride removal was reported elsewhere [63,64].

### 4.4. Effect of Coexisting Ions

**Figure 9:**
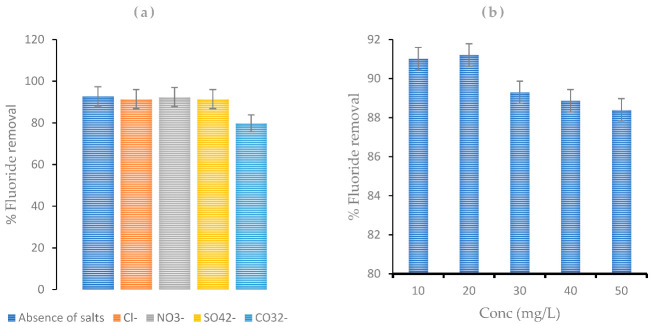
Effect of (**a**) co anions (0.25 g at 10 mg/L of fluoride ion with 100 mg/L of the respective individual coexisting anion); (**b**) co anion composite concentration on fluoride sorption by CNF-AgMgOnHaP composite (adsorbent dose: 0.25 g, 10 mg/L, contact time 10 min at 250 rpm).

The possible interference of coexisting ions (such as Cl^−^, NO_3_^−^, CO_3_^2−^, and SO_4_^2−^), along with fluoride for the active sorption sites in the sorbent material, was examined, and the results are presented in Figure 9a,b. Figure 9a illustrates the variation in F^−^ removal efficiency by the adsorbent with the existence of co anions in water. The result showed that the presence of anions such as Cl^−^, NO_3_^−^, and SO_4_^2−^ had a slight effect on fluoride sorption by CNF-AgMgOnHaP, with NO_3_^−^ interfering the least when compared with the anion free water. However, the competitiveness of CO_3_^2−^ appeared to have a significant effect in the F^−^ uptake for surface binding sites, leading to a reduction in % fluoride removal from ~93% to ~80%. In addition, the effect of co anion composite concentrations (10–50 mg/L), as a function of percentage fluoride removal by the CNF-AgMgOnHaP composite, is depicted in Figure 9b. Evidently, from the plot (Figure 9b), at higher composite anion concentrations, the potential affinity for fluoride by the sorbent reduces. This may be because of the strong hydrolysis contribution by stable CO_3_^2−^ ions surface complex formation, which hinders fluoride ions being sorbed by the CNF-AgMgOnHaP composite.

### 4.5. Effect of Initial Concentration and Adsorption Isotherm

**Figure 10:**
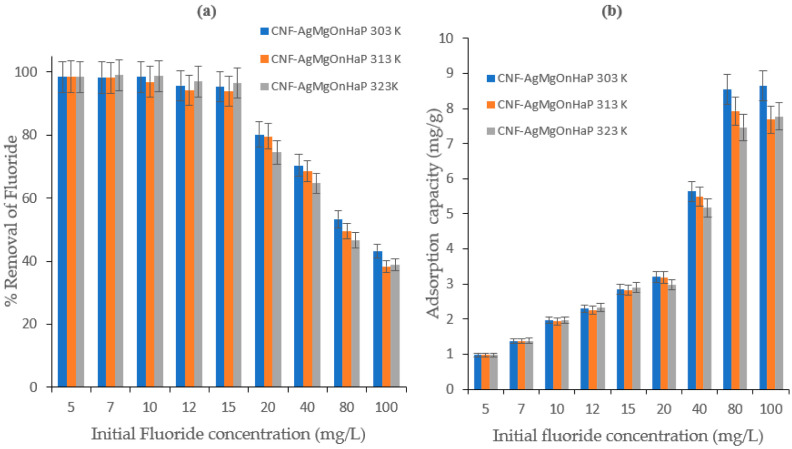
Effect of initial fluoride concentration on CNF-AgMgOnHaP adsorbent at different temperatures.

The rate of fluoride uptake by the CNF-AgMgOnHaP composite as a function of initial fluoride concentrations (5–100 mg/L) at different temperatures (303, 313, and 323 K) are shown in Figure 10a,b. As depicted in Figure 10a, the % F^−^ removal is found to decrease with increasing initial adsorbate concentration. This may be due to the reduction in active binding sites on the CNF-AgMgOnHaP adsorbent surface leading to its saturation by the sorbed fluoride ion. Furthermore, in contrast to Figure 10a, the F^−^ sorption capacity increased gradually as the fluoride concentration increased from 5 to 100 mg/L (Figure 10b). This suggests that, as the concentration increases, the driving force for overcoming the mass transfer resistance between the adsorbate in solution and adsorbent phases increases in the liquid–solid adsorption system [64].

To determine the adsorption interaction and mechanism of the F^−^ ions on the CNF-AgMgOnHaP composite surface, the nonlinear forms of Langmuir [65,66] (Equations (3) and (5)), as well as the linear form of Dubinin–Radushkevich [67] (Equations (6)–(9)) isotherm models were used to evaluate the adsorption process.

These equations were used to simply calculate the adsorption parameters because of the usefulness of their model parameters, their clarity, and easy interpretability, as well as to reduce their respective error functions [57,68].

The nonlinear forms of the Langmuir adsorption isotherm model [69] assume a monolayer of F^−^ sorption onto the surface of CNF-AgMgOnHaP containing a finite number of uniform adsorption binding sites without the migration of adsorbed molecules on the surface at a fixed temperature, and is given as:

(3) $$q_{e}=\frac{Q_{m}K_{L}C_{e}}{1+K_{L}C_{e}}$$

where *Q_m_* (mg/g) is the maximum adsorption capacity, which assumes the complete monolayer of an adsorbent, *C_e_* (mg/L) is the adsorbate equilibrium concentration, $q_{e}$ (mg/g) is the amount of F^−^ ion adsorbed at equilibrium, and *K_L_* (L/mg) is the Langmuir adsorption equilibrium constant.

The fundamental characteristics of the Langmuir isotherm model can also be calculated in terms of a dimensionless parameter called the separation factor, *R_L_* [68] (Equation (4)), denoted as:

(4) $$R_{L}=\frac{1}{1+k_{L}C_{i}}$$

where *C_i_* (mg/L) is the initial fluoride concentration and *K_L_* is the Langmuir equilibrium constant. The *R_L_* value is useful in determining if a sorption process is irreversible (*R_L_* = 0), linear (*R_L_* = l), favorable (0 < *R_L_*< 1), or unfavorable (*R_L_* > l).

The Freundlich adsorption isotherm [66], which is an empirical model describing the surface heterogeneity of the CNF-AgMgOnHaP sorbent through a multilayer adsorption system, was also applied. It is expressed below as:

(5) qe=KFCe1n

where $q_{e}$ (mg/g) is the equilibrium adsorption capacity of the CNF-AgMgOnHaP adsorbent, *C_e_* (mg/L) is the equilibrium concentration, and *K_F_* [(mg/g)/(mg/L)^n^] and 1/*n* are empirical Freundlich constants describing sorption capacity and intensity parameter, respectively. *K_F_* characterizes the strength of adsorption, while *n* indicates the magnitude of the adsorption driving force or the surface heterogeneity [67]. The adsorption isotherm is favorable when 0 < 1/*n* < 1, unfavorable when 1/*n* > 1, and irreversible when 1/*n* = 1.

The equilibrium adsorption results were also fitted into the linearized form of the Dubinin Radushkevich isotherm model [68], which accounts for the effects of the porous nature of the CNF-AgMgOnHaP adsorbent and the mean free energy (E) of the sorption process. The D-R equations are shown below.

(6) $$q_{e}=q_{max\;}exp\left(-\beta_{D}\varepsilon^{2}\right)$$

(7) $$\varepsilon =RT\;ln\left[1+\frac{1}{C_{e}}\right]$$

The energy *E* (kJ/mol) of F^−^ sorption from Dubinin–Radushkevich is

(8) $$E=\frac{1}{\sqrt{2\beta_{D}}}$$

The general linearized form of the D-R is in Equation (9):

(9) $$ln\;q_{e}=ln\;q_{max}-\beta_{D}\varepsilon^{2}$$

where $q_{max}$ (mg/g) is the CNF-AgMgOnHaP adsorption capacity; $\beta_{D}$ (mol^2^/kJ^2^) is the activity coefficient constant related to mean sorption energy; *ε* is the Polanyi potential; and *E* (kJ/mol) is the mean adsorption energy. The slope of the plot of ln $q_{e}$ against *ε*^2^ gives $\beta_{D}$ and the intercept gives the adsorption capacity $q_{max}$ of CNF-AgMgOnHaP. If the magnitude of *E* is between 8 and 16 kJ/mol, the adsorption process occurred via chemisorption, while for values where *E* < 8 kJ/mol, the adsorption process is physisorption.

**Figure 11:**
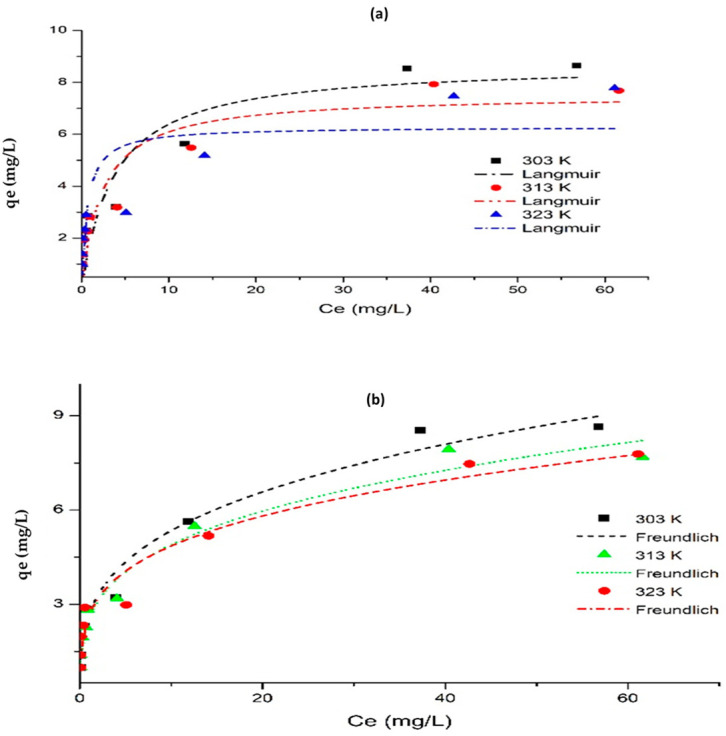
Nonlinear isotherms by (**a**) Langmuir and (**b**) Freundlich methods, for the sorption of fluoride by CNF-AgMgOnHaP composite at 303 K, 313 K, and 323 K.

Figure 11 and Table 3 show the adsorption isotherms plots with the corresponding calculated parameters of the sorbed F^−^ on CNF-AgMgOnHaP composite at different temperatures.

As shown in Table 3, the respective values of *Q_m_* and *K_F_* from the nonlinear plots decrease as the temperature increases, an indication that the adsorption of F^−^ by the CNF AgMgOnHaP composite is unfavorable at higher temperatures. The Langmuir constant (*K_L_*) values increase with temperature changes, showing the existence of an electrostatic attraction between the F^−^ and the CNF AgMgOnHaP surface [69]. The obtained *R_L_* values lying between 0 and 1 also confirm the favorability of the adsorption activity across all the operating temperatures. In addition, since *n* lies between 1 and 10 as shown in Table 3, the physical adsorption of F^−^ onto the adsorbent is demonstrated. It is important to note that the experimental maximum adsorption capacities were observed to be similar to the calculated Langmuir maximum adsorption capacities (*Q_m_*) for all the temperatures. In addition, the adsorption energies, *E* (kJ/mol) values, obtained by the linear D-R model generally increased as the temperature increased (Table 3). These values suggested that the physical adsorption mechanism was involved in the F^−^ sorption process. Consequently, based on the higher correlation coefficients (*Adj. R*^2^) and lower Chi-square (*χ*2) values comparison (Table 3), it was found that the fluoride sorption onto the CNF-AgMgOnHaP was best described by the Freundlich isotherm model across all the operating temperatures, compared to other models. This suggested that the dominant adsorption mechanism on the adsorbent surface was through multilayer binding sites.

### 4.6. Adsorption Kinetic

To evaluate the mechanism and time dependence kinetic parameters of F^−^ sorption by the CNF-AgMgOnHaP surface, the contact time experimental data (Figure 6) were simulated and fitted to both reaction and diffusion based kinetic models.

The Lagergren [70] pseudo-first-order model can be used for a simple sorption process and its linear equation is stated in Equation (10):

(10) $$log\left(q_{e}-q_{t}\right)=\;log\;q_{e\;}-\frac{K_{1}}{2.303}\;t$$

The linearized pseudo-second-order (Equation (11)) model, which is normally used to describe cation exchange and chemisorption reaction mechanisms [71], was also employed and is represented by Equation (11):

(11) $$\frac{t}{q_{t}}=\frac{1}{K_{2}q_{e}^{2}}+\frac{t}{q_{e}}$$

The linear form of the intraparticle diffusion model [72] given in Equation (12) was also used to describe the transfer of the solute in the solid/liquid system.

(12) $$q_{t}\;=\;k_{i}t^{0.5}\;+\;C$$

where *q_e_* and *q_t_* are the amounts of adsorbate uptake per mass of CNF-AgMgOnHaP adsorbent (mg/g) at equilibrium and at time *t* (min), respectively; with $K_{1}$*,* $K_{2}$ and *K_i_* (min^−1^) representing the rate constant of the pseudo-first-order, pseudo-second-order, and intraparticle diffusion rate constant, respectively.

The normalized standard deviation (S.D. (%)) usually used to describe the applicability of the kinetic model in the fluoride sorption process on the adsorbent was determined using Equation (13).

(13) $$S\;.D.\;\left(\%\right)=100\sqrt{\frac{\sum \left[\frac{q_{e}exp-q_{e}cal}{q_{e}exp}\right]2}{N-1}}$$

where *N* is the number of data points, and *q_e_, exp*_,_ and *q_e_, cal* (mg/g) are the experimental and the calculated equilibrium sorption capacity, respectively.

The calculated corresponding adsorption kinetic parameters from the three different model plots for fluoride sorption are summarized in Table 4. Based on the higher correlation coefficient (*R*^2^) and the lowest S.D. and root mean square error (RMSE) values obtained regarding reaction based pathways, the pseudo-second-order model was the most suitable model and best describes the fluoride sorption behavior on the adsorbent surface. This confirmed that the reaction rate was rapid, as observed in Figure 6. This is an indication that the reaction mechanism may have been controlled by chemisorption, which involved the sharing or exchange of ions between the CNF-AgMgOnHaP adsorbent and the sorbed F^−^. This was supported by the XPS (Section 4.8) and pH at various sorbate sorbent pH values (Section 4.3). Equally, the intraparticle diffusion models parameters (Table 4) further showed that the overall kinetic models might not only depend upon the chemical adsorption process but were also governed by an equilibrium diffusion mechanism, through a mass transfer of the adsorbate molecules from the external surface through the pores of the adsorbent [73].

### 4.7. Thermodynamics

**Figure 12:**
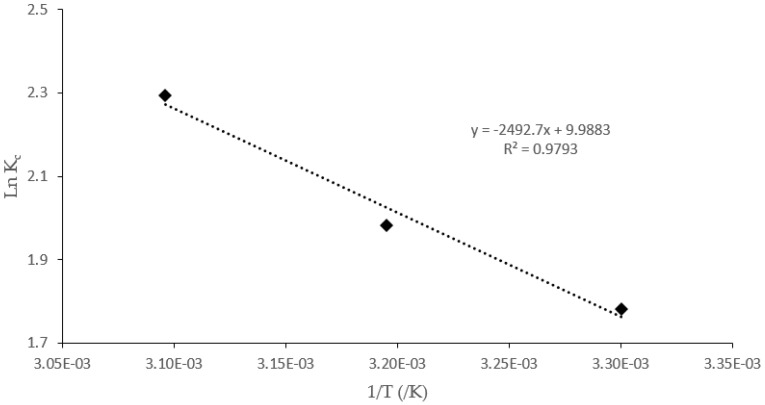
Here, ln *K_c_* as a function of reciprocal adsorption temperatures.

The thermodynamic parameters (Table 5), such as standard enthalpy change (∆*H°*), standard free energy (∆*G°*), and standard entropy change (∆*S°*), were evaluated from the experimental sorption data (303–323 K). These parameters were obtained from the plot of 490 1/T vs. ln *K_c_* (Figure 12) using the following equations (Equations (14)–(17));

(14) $$\Delta G{}^{\circ}=-RT\;ln\;K_{c}$$

(15) $$\;K_{c}=q_{e}/C_{e}$$

(16) $$lnK_{c}=-\frac{\Delta H{}^{\circ}}{RT}+\frac{\Delta S{}^{\circ}}{R}$$

(17) ΔG°=ΔH°−TΔS°

where *R* (8.314 J mol^−1^ K^−1^) is the gas constant, and *T* is the absolute temperature (K). The equilibrium constant values, *K_c_*, were obtained by plotting *q_e_/C_e_* against *C_e_* and extrapolating to zero [74,75].

The calculated thermodynamic parameters are listed in Table 5. The positive value of Δ*H°* is an indication that the fluoride sorption process stimulation occurred at a reasonably higher temperature and is, thus, endothermic [76]. This was further displayed by the decrease in the values of *Q_m_* and *K_F_*, respectively, as well as the increased *K_a_* values from the nonlinear isotherm parameters (Table 3) as the temperature increases. The values of ∆*G°* obtained are all negative across the temperature range, which implies that the fluoride adsorption process occurred favorably and spontaneously with minimal requirements of the adsorption and activation energies [68]. The positive ∆S° values suggest that the F^−^ sorption phenomenon was governed by the increasing randomness of the F^−^ ions at the CNF-AgMgOnHaP adsorbent–solution interface. 

### 4.8. Surface Chemistry

**Figure 13:**
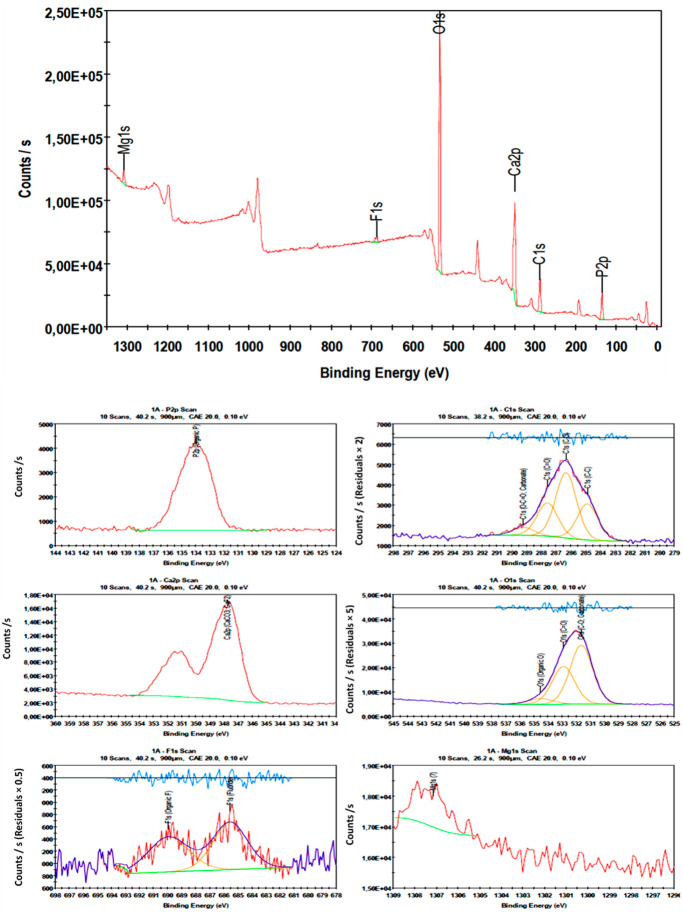
Wide scan XPS spectra of CNF-AgMgOnHaP adsorbent after fluoride adsorption.

Figure 13 shows the wide XPS spectral elemental analysis, whose peaks fitted according to the surface composition and chemistry of the fluoride sorbed CNF-AgMgOnHaP adsorbent. The fundamental elements of the adsorbent, as displayed by the XPS spectrum, include carbon, oxygen, calcium, phosphorus, magnesium, fluorine, and traces of silver content. As shown in Figure 13, the C1s (287.1 eV) spectra are resolved into four gaussian peaks assigned to C-C (284.9 eV), the C-O contribution of -OH groups (286.3 eV), C=O (287.6 eV), and O-C=O (289.2 eV) bonding states [77,78], associated with the cellulose nanofiber layer within the composite formation. The peaks of O 1s and Mg 1s, which appeared between 531 and 535 eV (deconvoluted into three major peaks) and 1307.2 eV (single peak), respectively, are a representation of lattice O atoms bonded with carbonyl, Mg atoms, hydroxyl groups, and adsorbed water [78]. The presence of 2p (134.0 eV) and Ca 2p (347.7 eV) were attributed to organic P and Ca-C=O interaction for calcium phosphates in the hydroxyapatite. The fewer related peaks at ~367.8 eV (two lines) corresponded to the characteristic peak of Ag NPs, confirming its existence within the composite. The appearance of an F 1s peak at 685 eV, which was resolved into two peaks at 685.5 and 689.9 eV, attributed to inorganic and organic fluoride, respectively, indicates F^−^ ions being bound to the CNF-AgMgOnHaP adsorbent. This was further supported by the possible Ca-F bond, as shown by the Ca 2p (347.7 eV). Thus, the XPS, EDS, FTIR, and solution pH results clearly show that the surface mechanisms of fluoride removal by the CNF-AgMgOnHaP composite could have occurred via ligand exchange and electrostatic attraction between F^−^ and the OH^−^, Mg^2+^ or Mg-OH^+^ and Ca-OH^+^ species at the sorbent–sorbate solution interface.

### 4.9. Regeneration and Reusability of CNF-AgMgOnHaP Adsorbent

**Figure 14:**
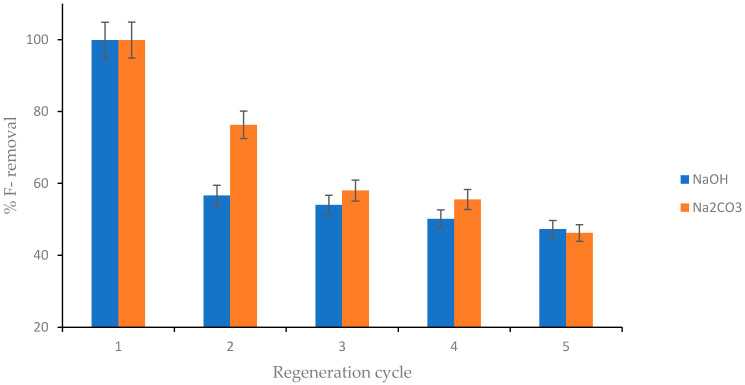
Percent fluoride removal as a function of defluoridation cycle using 0.01 M NaOH and 0.1 M Na_2_CO_3_ as regenerates (initial fluoride concentration: 10 mg/L, volume of solution: 50 mL, adsorbent dosage: 0.25 g contact time: 30 min at 250 rpm).

Figure 14 depicts the reusability trend against the percent fluoride removal from an aqueous solution at various cycles of defluoridation (1–5) with an adsorbent dose of 0.25 g/50 mL at 25 ± 3 °C. This was carried out based on the analysis of the effects of coexisting anions, which suggested that the capacity of the CNF-AgMgOnHaP adsorbent to remove fluoride in a solution was low in alkaline solution. Therefore, different concentrations of NaOH and Na_2_CO_3_ were used in this study. As shown in Figure 14, fluoride removal by a sorbent regenerated with NaOH was observed to be very low when compared to that regenerated with Na_2_CO_3_. For both regenerants, the percentage of fluoride removal by the composite decreases (~48% for NaOH and 46% Na_2_CO_3_) with an increasing regeneration cycle. It is important to note that the type of surface interaction between the regenerants and the adsorbent determines the reusability property; hence, the provision of better regeneration capability by the adsorbent may have been through electrostatic and chemisorption interactions between the adsorbent and F^−^, which occur naturally in the groundwater [79,80]. Consequently, the regenerated CNF-AgMgOnHaP adsorbent possesses a greater economic potential in the removal and recovery of fluoride ions, even in the presence of a limiting solution parameter.

### 4.10. Antibacterial Activity of the CNF-AgMgOnHaP Adsorbent

**Figure 15:**
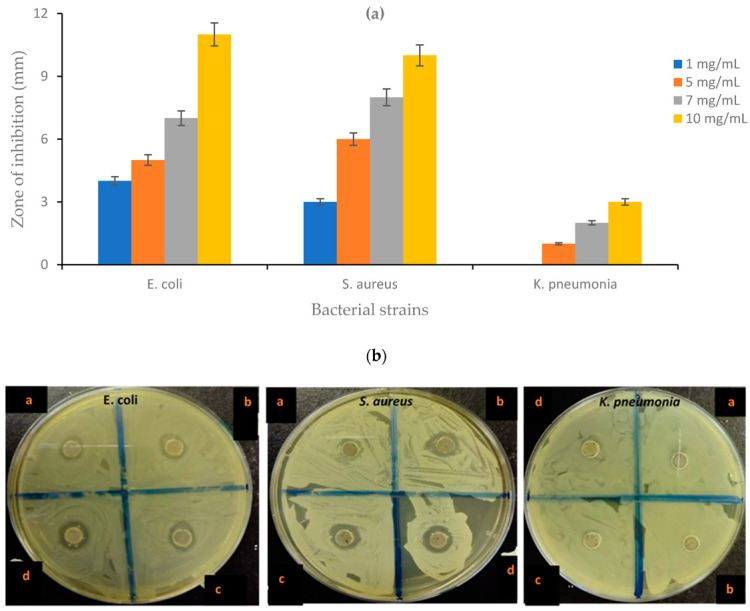
(**a**) Graphical representation and (**b**) photographic plate of antibacterial activity by the CNF-AgMgOnHaP composite against *E. coli*, *S. aureus*, and *K. pneumonia* at different concentration ranges.

Figure 15a,b displayed the observed antibacterial property of the CNF-AgMgOnHaP adsorbent through the values exhibited by the zone of inhibition against Gram-negative and Gram-positive bacterial strains. The diameter of the zone of inhibition was found to increase with an increase in the concentration of the adsorbent (Label: a, b, c, d depicting 1, 5, 7, and 10 mg/L, respectively) (Figure 15b). The observed zone of inhibition showed that the CNF-AgMgOnHaP adsorbent possesses antibacterial activity against all the bacterial strains. However, this antibacterial property varies, with higher antimicrobial potency observed towards *E. Coli* compared to *S. aureus* and *K. pneumonia*.

The antibacterial potency observed by this material depends on several factors, including the synergistic effects of Ag-MgO within the synthesized CNF-AgMgOnHaP composite. It has been established that MgO and Ag based materials have excellent broad spectrum, potent antimicrobial properties and are easily impregnated into cellulosic materials as a stabilizing agent [81,82,83]. The antibacterial property of the CNF-AgMgOnHaP adsorbent may be due to the diffusion of Ag-MgO nanoparticles within the adsorbent through the release of metal ions disrupting the cell wall structure of the bacteria genome in producing intracellular reactive oxygen species (ROS), resulting in microbial cell death [18,84,85]; the Ag-MgO nanocomposite antibacterial activity may also depend on the reducing and capping agents used in the synthesis route, as well as the size and surface properties of the composite [38,86,87]. It is important to emphasize that the Ag ions release, though in low concentration, was affected by the time and rate of dissolution; metal ions release increases as time progresses. Therefore, the adsorbent presents specific antibacterial activity against all the bacteria strains.

## 5. Comparative Analysis

To prove the systemic integrations of the polymers cum nanoparticles and the role of the active functional groups within the composite structures, the CNF-AgMgOnHaP was compared with similar sorbent materials reported in the literature (Table 6). As shown in Table 6, the CNF-AgMgOnHaP composite has an excellent performance and advantages, such as its disinfection property and highest fluoride removal efficiency, which makes it a suitable material for household water treatment for improved socioeconomic development.

## 6. Conclusions

A CNF-AgMgOnHaP composite was successfully biosynthesized through the impregnation and dispersion of Ag-MgO and nHap nanoparticles by a simple hydrothermal method. The structural morphology and optical properties provided evidence showing the aggregation of nanoparticles on the biopolymeric cellulose fiber matrix, after modification. The increased surface area of the CNF-AgMgOnHaP composite by BET analysis showed the availability of numerous binding functional groups within the matrix, which aligned with other spectro-analytical results. The sorption of fluoride by the adsorbent was found to strongly depend on the different sorption conditions, with the optimum adsorption conditions determined at 10 min (25 ± 3 °C), 0.25 g adsorbent dose, and pH 5, with a maximum defluoridation capacity of 8.712 mg/g at 303 K. The sorbate–sorbent interaction was well documented by adsorption isotherms and kinetic models and based on the higher correlation coefficients (*R*^2^) and lower Chi-square (*χ*2) values comparison; it was found that the fluoride sorption onto the CNF-AgMgOnHaP was best described by the Freundlich isotherm model across all the operating temperatures. Based on the higher correlation coefficient (*R*^2^) and the lowest S.D. and RMSE values obtained with regards to reaction based pathways, the pseudo-second-order model was the most suitable model and best describes the fluoride sorption behavior on the adsorbent surface. However, the overall kinetic results indicated that the mechanisms not only depend on the chemical adsorption process but were also governed by the mass transfer of the adsorbate molecules from the external surface through the pores of the adsorbent. The thermodynamic parameters revealed that the adsorption process of F^−^ onto CNF AgMgOnHaP was endothermic and spontaneous. The synthesized composite also provides some antibacterial activity against both Gram-negative and Gram-positive water bacteria strains. Consequently, the synthesized CNF AgMgOnHaP composite can be used as a suitable and viable adsorbent in the simultaneous removal of fluoride and pathogen in drinking water.

## Figures and Tables

**Table 1:** Optimization of CNF % weight in the CNF-AgMgOnHaP adsorbent for F^−^ removal.

CNF (*w/w* %)	Average Equilibrium Fluoride Concentration (*C_e_*) (mg/L)	% Fluoride Removal
10	3.307	66.93
30	1.364	86.36
50	0.476	95.24
70	0.343	96.57
100	0.096	99.04

**Table 2:** BET Surface analysis parameters of CNF-AgMgOnHaP adsorbent.

BET Parameter	Methods	Values
**Surface area**	BET surface area	160.17 m^2^/g
	Langmuir surface area	220.45 m^2^/g
	t-Plot external surface area	154.71 m^2^/g
**Pore Area**		
Micropore area	t-Plot micropore area	5.46 m^2^/g
Mesopore area	BJH adsorption	196.12 m^2^/g
	BJH desorption	223.89 m^2^/g
**Pore volume**		
Micropore volume	Single point adsorption	0.38 cm^3^/g
	Single point desorption	0.38 cm^3^/g
Mesopore volume	BJH adsorption	0.39 cm^3^/g
	BJH desorption	0.40 cm^3^/g
**Pore size**		
Adsorption average pore width	4 V/A by BET	9.55 nm
Desorption average pore width	4 V/A by BET	9.53 nm
Mesopore size	BJH adsorption	7.91 nm
	BJH desorption	6.91 nm

**Table 3:** Isotherm parameters for the sorption of fluoride by CNF-AgMgOnHaP composite.

	Temperature (K)	303	313	323
**Non-Linear**	**Langmuir isotherm**			
	*Q_m_* (mg/g)	8.715	7.52	6.286
	*K_L_* (L/mg)	0.275	0.426	1.566
	*R_L_*	0.27	0.19	0.06
	*Adj. R* ^2^	0.813	0.845	0.709
	*Red. χ*2	1.632	1.085	1.879
	*RSS*	11.42	7.59	13.16
	**Freundlich isotherm**			
	*K_F_* [(mg/g)/(mg/L)^n^]	2.686	2.547	2.671
	*n*	3.345	3.519	3.853
	*Adj. R* ^2^	0.973	0.973	0.952
	*Red. χ*2	0.308	0.188	0.308
	*RSS*	2.158	1.318	2.158
**Linear**	**Dubinin–Radushkevvich**			
	*β_DR_* (mol^2^/kJ^2^)	4.00 × 10^−8^	3.00 × 10^−8^	3.00 × 10^−8^
	*q_max_* (mg/g)	5.046	4.448	4.804
	*E* (kJ/mol)	3.535	4.083	4.083
	*R* ^2^	0.749	0.698	0.759

**Table 4:** Kinetic model parameters for adsorption of F^−^ by CNF-AgMgOnHaP.

Model	Values
**Pseudo-first order**	
*q_cal_* (mg/g)	0.84
*k_1_* (min^−1^)	0.23
*R* ^2^	0.89
*RMSE*	0.24
*S. D.* (%)	63.53
**Pseudo-second order**	
*q_cal_* (mg/g)	2.24
*k_2_* (g/min mg)	0.67
*R* ^2^	0.99
*RMSE*	0.082
*S. D.* (%)	18.21
**Intraparticle diffusion**	
*C*_1_ (mg/g)	1.64
*C*_2_ (mg/g)	1.62
*C*_3_ (mg/g)	1.9
*K_i1_* (mg/g.min^0.5^)	0.16
*K_i2_* (mg/g.min^0.5^)	0.14
*K_i3_* (mg/g.min^0.5^)	0.064
*R* ^2^_1_	1
*R* ^2^_2_	0.99
*R* ^2^_3_	1

**Table 5:** Fluoride adsorption thermodynamic parameters.

Temperature (K)	Δ*H°* (KJ/mol)	Δ*S°* (J/mol K)	Δ*G°* (kJ/mol)
	20.73	83.05	
303			−4.44
313			−5.27
323			−6.10

**Table 6:** Comparative analysis of CNF-AgMgOnHaP to other materials.

Sorbent Materials	F^−^ Sorption Capacity (mg/g)	Optimized Condition	Microbial Removal Potential	Reference
HaP nanorods	1.49	3 h. pH 7; 7 g/L	Nil	[88]
Sawdust raw	1.73	pH 6, 0.5 g/25 mL	Nil	[89]
Cellulose–hydroxyapatite	4.2	pH 6.5.	Nil	[90]
Ag/MgOnHaP	2.15	60 min; pH 6; 0.3 g	Yes	[26]
AgMgOnHaP@CSn	6.86	40 min; pH 7; 0.25 g	Yes	[28]
CNF-AgMgOnHaP	8.71	0.25 g; 10 min; pH 5	Yes	This study

## Data Availability

Not applicable.
